# Hernia among Patients Admitted to the Department of Surgery of a Tertiary Care Centre: A Descriptive Cross-sectional Study

**DOI:** 10.31729/jnma.8085

**Published:** 2023-03-31

**Authors:** Kishor Kumar Deo, Reshika Shrestha, Saroj GC, Sujata Maharjan, Aishwarya Shrestha, Asmita Neupane

**Affiliations:** 1 Department of Surgery, National Academy of Medical Sciences, Mahaboudha, Kathmandu, Nepal; 2 Maharajgunj Medical Campus, Maharajgunj, Kathmandu, Nepal; 3 Bajrabarahi Chapagaun Hospital, Bajrabarahi, Lalitpur, Nepal; 4 Shahid Gangalal National Heart Center, Bansbari, Kathmandu, Nepal; 5 Kathmandu Medical College and Teaching Hospital, Sinamangal, Kathmandu, Nepal

**Keywords:** *hernia*, *inguinal hernia*, *prevalence*, *surgery*, *umbilical hernia*

## Abstract

**Introduction:**

Hernia is one of the most common surgery-requiring conditions. Despite this, hernia still needs to be studied in more detail. The main objective of the study was to find out the prevalence of hernia among patients admitted to the Department of Surgery of a tertiary care centre.

**Methods:**

A descriptive cross-sectional study was conducted among patients admitted to the Department of Surgery of a tertiary care centre from 1 July 2021 to 31 December 2022. Ethical approval was obtained from the Institutional Review Committee (Reference number: 202/2079/80). The patient admitted to the Department of Surgery during the study period was included and those with incomplete data were excluded. A convenience sampling method was used. Point estimate and 95% Confidence Interval were calculated.

**Results:**

Among 3236 patients, the prevalence of hernia was 749 (23.14%) (21.69-24.59, 95% Confidence Interval). The inguinal hernia was the most common type found in 574 (77.25%), followed by an umbilical hernia in 64 (8.61%). A total of 79 (10.55%) had comorbidity among patients with hernia.

**Conclusions:**

The prevalence of hernia in our study was found to be higher than in other studies done in similar setting. Easily accessible health facilities, competent primary surgical care and health education should be taken into account by the policymakers to reduce the morbidity and mortality of this condition.

## INTRODUCTION

Hernia is an outpouching which may contain parts of viscera.^1^ Hernia repair is one of the most common surgical procedures performed annually on millions of patients worldwide. Groin hernia repair is globally performed on more than 20 million patients per year.^2^ The outcome in a developing nation depends on a number of variables, including accessibility, national healthcare systems, the resources available, and the surgeon's skill.^3^

In Nepal, the estimated number of people with groin masses is 310,000 and nearly 66,000 males with soft/reducible groin masses are in need of evaluation. Nearly 40% of people with hernia reported disability.^4^

So, the burden of a hernia should be evaluated to improve the quality of life of people living with a hernia and increase the quality of life of the affected patients.

This study aimed to find out the prevalence of hernia among patients admitted to the Department of Surgery of a tertiary care centre.

## METHODS

A descriptive cross-sectional study was conducted among patients admitted to the Department of Surgery of the National Academy of Medical Sciences, Kathmandu, Nepal. Ethical approval was obtained from the Institutional Review Committee (Reference number: 202/2079/80). Data were collected from the hospital record section from 1 July 2021 to 31 December 2022. Patients who were admitted to the Department of Surgery during the aforementioned study period were included and those with incomplete hospital record data were excluded from the study. A convenience sampling method was used. The sample size was calculated using the following formula:


$$\begin{array}{ll} \text{n} & ={\text{Z}}^{2}\times \frac{\text{p}\times \text{q}}{{\text{e}}^{\text{2}}}\\ & =1.96^{2}\times \frac{0.50\times 0.50}{0.02^{2}}\\ & =2401 \end{array}$$

Where,

Z = 1.96 at a 95% Confidence Interval (CI)
p = prevalence taken as 50% for maximum sample size calculation
q = 1-p
e = margin of error, 2%

Thus, the calculated minimum required sample size was 2401. However, 3236 patients were taken for the study.

The details of patients with hernia were recorded in a performed proforma which included age, sex, types of hernia, and comorbidities. The diagnosis was made on the basis of history and clinical examination. Ultrasonography was also done in doubtful cases. The data was collected from the hospital record books.

Data were entered in Microsoft Excel 2016 and analysed using IBM SPSS Statistics 16.0. Point estimate and 95% CI were calculated.

## RESULTS

**Figure 1:**
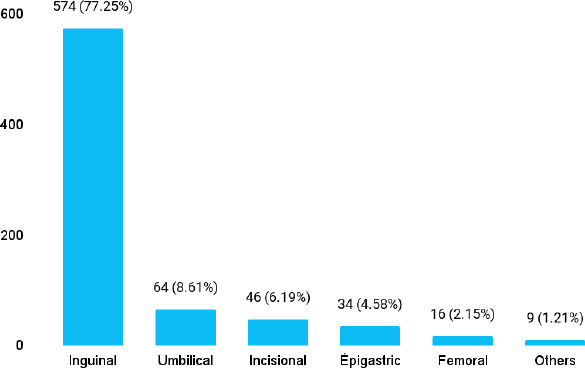
Types of hernia (n= 743)

Out of 3236 patients, the hernias were seen in 749 (23.14%) (21.69-24.59, 95% CI). The inguinal hernia was seen in 574 (77.25%) and umbilical hernia in 64 (8.61%) patients (Figure 1).

There were 643 (86.54%) males and 100 (13.45%) females. The mean age of the patients was 52.64±17.87 years (range: 14-98). The male:female ratio of overall hernia patients was 6.43:1 and that for the inguinal hernia was 23.95:1, the umbilical hernia was 1.78:1, the incisional hernia was 1:2.06, the epigastric hernia was 2.77:1 and the femoral hernia was 1:1.66. Among patients with inguinal hernia, 551 (95.99%) were male and 119 (20.73%) were in the age group of 65-74 years (Table 1).

**Table 1:** Sex and age-wise distribution of patients with hernia (n= 743).

Variable	Inguinal hernia n (%)	Umbilical hernia n (%)	Incisional hernia n (%)	Epigastric hernia n (%)	Femoral hernia n (%)
**Sex**					
Male	551 (95.99)	41 (64.06)	15 (32.61)	25 (73.53)	6 (37.50)
Female	23 (4.01)	23 (35.94)	31 (67.39)	9 (36.47)	10 (62.50)
**Age group (years)**					
0-17	14 (2.44)	-	-	-	1 (6.25)
18-24	35 (6.10)	3 (4.69)	-	2 (5.88)	-
25-34	60 (10.45)	10 (15.63)	6 (13.04)	5 (14.71)	3 (18.75)
35-44	75 (13.07)	9 (14.06)	8 (17.39)	7 (20.59)	-
45-54	91 (15.85)	17 (26.56)	15 (32.61)	11 (32.35)	2 (12.50)
55-64	119 (20.73)	10 (15.63)	9 (19.57)	7 (20.59)	4 (25)
65-74	103 (17.94)	11 (17.19)	6 (13.04)	2 (5.88)	3 (18.75)
75-84	61 (10.63)	3 (4.69)	2 (4.35)	-	2 (12.50)
>85	16 (2.79)	1 (1.56)	-	-	1 (6.25)
Total	574 (77.25)	64 (8.61)	46 (6.19)	34 (4.58)	16 (2.15)

A total of 79 (10.55%) had comorbidity with the most common being hypertension seen in 25 (3.34%), and diabetes mellitus in 13 (1.74%) patients (Table 2).

**Table 2:** Comorbidities among patients with hernia (n= 743).

**Comorbidities**	**n (%)**
Hypertension	25
DM	-3.34
Hydrocele	13
Chronic obstructive pulmonary disease	-1.74
Cholelithiasis	12
Others	-1.6

## DISCUSSION

In our study, the prevalence of hernia was 23.14%. A similar study done in Ethiopia showed a prevalence of 11.7% which was lower than in our study.^5^ Abdominal wall hernia among patients admitted to a tertiary care centre in India showed the burden of abdominal hernia to be 22%.^6^ A meta-analyis showed 7.7% as the pooled prevalence of inguinal hernia.^7^ A retrospective analysis of the National Center for Health Statistics in the US showed a 15.6% baseline prevalence of abdominal inpatient emergent hernia repaired from 2001 to 2010 over 10 years.^8^ The institution-based study done among adult patients visiting the surgical outpatient department at the University of Northwest Ethiopia showed the prevalence of external hernia among to be 11.7%.^5^ The prevalence of abdominal wall hernia was found in 20.9% of Russian residents.^9^ The age-standardized rate for inguinal hernia in men ranged from 1144 per 100,000 persons between ages 5 and 49 years and 2941 per 100,000 persons aged 50 years.^4^ There are still people who were not able to have surgery due to a lack of surgical services, fear or mistrust of the surgical system and inability to afford care in Nepal.^4^

A study done in the Department of Defense Military Health System Data Repository of the US among the paediatric population aged less than or equal to 17 years showed that Inguinal hernia repairs were the most common, comprising 63% of all repairs, followed by umbilical (30%), ventral (7%), and femoral (<1%) repairs.^10^ Our study showed that 14 (93.33%) cases were of inguinal hernia repair among the paediatric population. The proportion of other types of hernia in this population could not be demonstrated as the sample was less in comparison to the study done by large registries. The prevalence of inguinal hernia was 77.81%, the umbilical hernia was 5.93% and incisional hernia was 3.12.^5^ The overall prevalence of abdominal hernias was 11.7% in a study done in Northern Saudi Arabia with the most common cases being para-umbilical 33.9%, inguinal 27.3%, and umbilical in 20.8%.^11^ Another study done among Russian population showed umbilical hernias were found in 10.2%, groin hernias in 8.3%, and incisional in 2.4% of residents.^9^

The distribution of hernia was found to be increasing with the age up to a certain age and then showed a declining curve which is in contrast to a recent study which shows a bimodal distribution, with peaks around age 5 and after age 70.^12^ The incidence of inguinal hernia repair is lowest in early adulthood and rises until the incidence peaks between the age of 70 and 80 years for both genders.^13^ The incidences of inguinal hernia repair increase almost exponentially for men during their third decade and onward, whereas the corresponding incidence for women exhibits a slow increase with increasing age.^14^

In our study, the male-to-female ratio of inguinal hernia and umbilical hernia showed male preponderance whereas femoral hernia was found to be more in the female which was in coherence with the findings of another study in which the male-to-female ratio was 82:1 among inguinal hernias, and 1.71:1 among umbilical hernias.^6^ Our study showed that male was affected in 86.54% which contrast with the study done in the Russian population which showed 31.2% were men and 14.6% were women. A study done among cohorts of Eastern Uganda found the overall prevalence of groin hernia in men to be 9-4 percent.^15^

Comorbidities associated with inguinal hernia are male gender, low body mass index, Ehlers-Danlos syndrome, prostatomegaly, smoking, COPD, etc.^7,14^ The comorbidity present in a study showed prostatism in 15.93%, hypertension in 10.93% and DM in 3.12%.^6^ In contrast to the finding, this study showed only 3.34% of patients had hypertension and 1.74 had diabetes.

The study was descriptive so the association between type of surgery and recurrence rates, and an association of comorbidities with types of a hernia could not be explored. Systematic random sampling could have been used, however, we have included a large sample population to make it nearly representative of the target population. An analytical study can be done for further insight into the association of the variables in the context of the Nepalese population. Being a referral tertiary care centre, these cases were evaluated and managed accordingly with the best available resources in the country so this might have overestimated the prevalence of hernia. There is still a larger group of the population in Nepal who are currently in need of surgical evaluation for a hernia. So, a nationwide community-based study with proper methodological design is recommended for increasing generalizability.

## CONCLUSIONS

The prevalence of hernia in our study was higher than in other studies done in similar setting. The concerned stakeholders who were planning for strengthening the surgical care system should consider the provision of necessary healthcare logistics, finances, and human resources for the timely diagnosis and management of hernia.
